# On the emergence of metabolism: the evolution of proteins that powered life

**DOI:** 10.1098/rstb.2024.0090

**Published:** 2025-08-07

**Authors:** Vikas Nanda, Jan Siess, Bhanu Prakash Jagilinki, Robert M. Hazen, Shina Caroline Lynn Kamerlin, Ronald Koder, Dror Noy, Jonathan Silberg, F. Akif Tezcan, Rein Ulijn, Nathan Yee, Paul Falkowski

**Affiliations:** ^1^ Department of Biochemistry and Molecular Biology, Robert Wood Johnson Medical School, Rutgers: The State University of New Jersey, New Brunswick, NJ, USA; ^2^ Department of Chemistry and Biochemistry, University of Oklahoma, Norman, OK, USA; ^3^ Carnegie Institution for Science Earth and Planets Laboratory, Washington, DC, USA; ^4^ School of Chemistry and Biochemistry, Georgia Tech, Atlanta, GA, USA; ^5^ Department of Physics, The City College of New York, New York, NY 10031, USA; ^6^ Graduate Programs of Physics, Biology, Chemistry and Biochemistry, The Graduate Center of CUNY, New York, NY 10016, USA; ^7^ Department of Biotechnology, Migal Galilee Technology Center, Kiryat Shmona, Israel; ^8^ Tel-Hai Academic College, Upper Galilee, North District, Israel; ^9^ Biosciences Department, Rice University, Houston, TX, USA; ^10^ Department of Chemistry and Biochemistry, UCSD, La Jolla, CA, USA; ^11^ Advanced Science Research Center, The Graduate Center of CUNY, New York, NY 10016, USA; ^12^ School of Environmental and Biological Sciences, Rutgers: The State University of New Jersey, New Brunswick, NJ, USA; ^13^ IMCS, Rutgers: The State University of New Jersey, New Brunswick, NJ, USA

**Keywords:** oxidoreductase, metals, peptide, protein design, origin of life

## Abstract

Life is far from thermodynamic equilibrium. Hence, life must extract energy from the environment. On Earth, that energy is driven by networks of metabolic reactions in all cells which ultimately move electrons and protons (i.e. hydrogen atoms) across the planet. The origin of metabolism required the emergence and evolution of proteins. Proteins are nanometre-scale chemical machines—i.e. literal nanomachines which physically move. These nanomachines enable living systems to perform essential biochemical tasks from replication to metabolism; the latter being the engines of life. In all extant life on Earth, a small set of these nanomachines, called oxidoreductases, couple chemical energy from the environment with core redox reactions including photosynthesis, respiration and nitrogen fixation. The origins and emergence of complex life have been intimately tied with evolution of oxidoreductases. Here, using structure-based analyses, we describe the evolution of the protein catalysts in three biological epochs. First, thermodynamically driven polymerization reactions generated simple metal-binding peptides with specific sequences that catalysed core metabolic reactions. Second, these catalysts were incorporated in small structural ‘folds’. In the third epoch, these folds served as building blocks for extant, complex nanomachines.

This article is part of the discussion meeting issue ‘Chance and purpose in the evolution of biospheres’.

## 1. Introduction

The origin of life cannot be ‘discovered’ – it has to be ‘re-invented’.

Albert Eschemoser

### (a) Life is a multi-scale electrical circuit

All life on Earth is electric. By far, the largest source of energy powering life is light. Photosynthesis converts light from the Sun to electrical energy and protons, and in so doing, reduces inorganic carbon (e.g. CO_2_ and its equivalents) to organic molecules, that is molecules with a carbon atom covalently bonded to at least one hydrogen atom. In oxygenic photosynthesis, the source of electrons and protons is liquid water, while a by-product is molecular oxygen. In aerobic respiration, the oxidation of organic matter leads to a flux of electrons and protons (but not molecular hydrogen) through metabolic pathways (i.e. biological circuits) to reduce oxygen (the terminal electron acceptor) to water and CO_2_. The potential difference between the anode (organic matter; in its simplest form, sugars) and the cathode (molecular oxygen) provides over 1 eV of energy. In effect, these two processes evolved to become planetary metabolic half-cells. Indeed, all organisms are, on a global scale, half-cells powered by circuits connecting electron sources and sinks in the environment [1–3].

Ultimately the planetary-scale electronic circuit is driven by nanoscale reactions catalysed by a specific set of enzymes: the oxidoreductases. Enzymes are nature’s catalysts; they lower the activation barrier for reactions without being consumed. Specifically, the oxidoreductases move electrons over sub-nanometre distances to power catalytic reactions. Their behaviours are governed by the physics of electron transfer [4–6]. Groups of oxidoreductases form metabolic pathways. At the cellular scale, the current depends on the rate of catalysis and diffusion of substrates. In unicellular organisms, the flux of electrons proceeds through microbial communities [3]. In multicellular organisms, interactions between cells drive physiological circuits in the body. All of these metabolic processes combine biological and geochemical cycles into a unified global circuit across a planetary scale [3]. The flux of electrons from the proteins to a planetary scale directly connects life to the Earth as one, gigantic, electrical circuit.

There are two groups of oxidoreductases. One group of these enzymes transfer hydrogen atoms (protons or hydride atoms) in the form of reduced small organic molecules (e.g. NADPH, FADH, etc.). The evolution of coenzymes and this group of proteins has been reviewed elsewhere [7]. The second group moves electrons without direct coupling to protons using transition metals as cofactors. Both groups of oxidoreductases were essential in the emergence and evolution of metabolism. In this review, we focus on the latter.

The selection of the transition metals is very strongly coupled to the history of the oxidation state of Earth over time. The affinities of proteins for divalent metals, described by Irving & Williams, increase from Mn to Cu: Mn(II) < Fe(II) < Co(II) < Ni(II) ≈ Zn(II) < Cu(II) [8]. As such, one would expect copper to dominate the inorganic coordination chemistry of living systems. However, the availabilities of metal ions have changed considerably over Earth history. In the Archean eon, iron and manganese were highly soluble in aquatic systems, while copper, molybdenum and zinc were largely sequestered in sediments as sulfides [9]. Following the oxidation of the atmosphere about 2.4 Ga and later the ocean interior, iron and manganese largely precipitated as oxides, while molybdenum, copper and zinc became much more soluble [2,9,10]. Consequently, many of the earliest oxidoreductases contain iron, either as a free metal or more commonly in hemes and iron–sulfur clusters, while those that emerged later coordinate metals such as copper [11]. Although metal speciation in enzymes is largely driven by availability, the oxidoreductases maintain these ions in oxidation states far from thermodynamic equilibrium with the environment, providing a driving force for metabolism [9].

We assert that metabolism started at low electrochemical potentials and evolved to increasingly higher ones: the earliest forms of life were probably based on oxidation/reduction of hydrogen gas (e.g. 2H^+^ + 2e^−^ ⇌ H_2_; E′_red_ = −0.42 V at pH 7), which was readily available from volcanic sources [12].

Let us consider how this reaction is catalysed in the contemporary world. A series of enzymes, the hydrogenases, contain iron and/or nickel atoms. These proteins are extremely large, approximately 1000 amino acids in length. The chance of hydrogenase emerging spontaneously by random polymerization of the 20 amino acids used in life on Earth would be one in 20^1000^ or 10^2000^, that is to say virtually impossible. Much more likely was the spontaneous generation of small peptides, in the order of a few amino acids in length, which coordinated metals like iron and nickel, and catalysed the same reaction [13]. We postulate that these types of metallopeptide catalysts could have represented the earliest stage in the evolution of the proteins that power metabolism.

How did these metallopeptides evolve to become extremely complex nanomachines? Proteins often form complex structures, but these structures can be delineated into smaller subunits, or ‘folds’, which are found across the tree of life [14]. Regardless, single catalysts do not make a metabolic pathway—a sequence of enzymatically driven reactions with feedbacks. Larger proteins can accommodate both a primary active site and secondary sites for interacting with partner proteins in the complex cellular environment. These structures can further be combined into large protein nanomachines, allowing for allosteric interactions and dynamics that regulate catalysis. Complex protein assemblies can move electrons over large distances, connecting redox proteins with molecular wires or promoting electrical potentials across membranes. These nanomachine oxidoreductases are the diodes, capacitors and transistors that comprise metabolic circuits.

**Figure 1.**
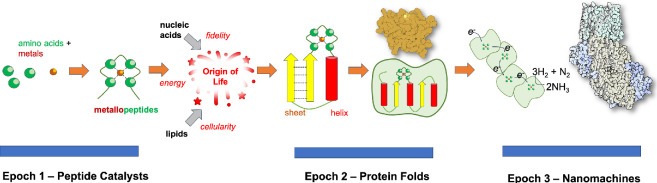
Emergence and complexification of metabolic proteins in three epochs. The foundations of metabolism started when a set of metallopeptides emerged through the spontaneous, thermodynamically driven selection of polymerizing amino acids bound to redox-active metal ions. These catalysts were the tipping point in the emergence of metabolism, giving rise to small metalloprotein folds and subsequent complex protein nanomachines.

Here we present this process of complexification as a series of three epochs: the first is the emergence of metallopeptides with amino acids as building blocks, the second is the formation of folds composed of secondary structures such as helices and sheets, and the third is the accretion of these folds into contemporary nanomachines (figure 1).

## 2. Epoch 1—the prebiotic emergence of metallopeptide catalysts

When complexed to transition metals, short peptides can perform redox catalysis, with the metal ions providing both stability through ligand–metal bonds and reactivity due to Lewis acidity [15]. In this section, we discuss how peptides, with defined sequences, i.e. the emergence of chemical information, could have preceded genetic replication due to thermodynamically driven chemical selection. As we will explain, laboratory models of early metallopeptides are surprising stable and reactive, suggesting that this first epoch of protein evolution produced environmentally resilient redox catalysts with the potential to kick-start life.

### (a) Spontaneous polymerization of peptides

The origin of life was a process of chemical complexification that must have occurred as a sequence of steps, from synthesis of the simplest organic building blocks to ever larger and more diverse molecular assemblages [16], ultimately providing the energy for replication [17–25]. A reasonable hypothesis is that at some stage of this process a subset of molecules formed an autocatalytic network (e.g. [26–28]), which evolved relatively rapidly via selection for more adaptive configurations through self-organization. The initial steps of prebiotic chemistry, the synthesis and accumulation of amino acids, sugars, lipids and other molecular building blocks via exogenous and endogenous processes [29], appears to be a deterministic aspect of many planets and planetary bodies. Inventories are documented both from carbonaceous meteorites and comets [30–34] as well as from organic synthesis in a variety of near-surface terrestrial environments [20,35–43].

Less certain are the mechanisms by which those simpler molecular components combined into large numbers of diverse oligomers and other configurations, a subset of which must have eventually displayed minimally functional properties that promoted molecular evolution. Given that the earliest functional peptides likely represent primitive enzymes, the pathways to prebiotic formation of peptide bonds under plausible prebiotic conditions are of special relevance [44]. In addition, the role of self-organization to drive the selection of certain ensembles of peptides from a complex sequence space is important to understand [45,46], i.e. how uncontrolled diversification (combinatorial explosion) is avoided. Pathways to prebiotic polymerization of amino acids without activating agents have been the subject of numerous prior investigations [47–49].

Four plausible prebiotic environmental attributes, singly or in combination, have been particularly successful in assembling polymers from amino acids or dipeptides.

(1) *Mineral surfaces*: Mineral surfaces have been invoked for a wide range of prebiotic processes, including as reactants, catalysts, templates and protective environments for organic molecules [50–54]. Indeed, peptide bond formation has been studied on a variety of mineral surfaces, including clays [55–59], hydroxyapatite [47–49,60], framework silicates [61–64], hydroxides [65–67] and sulfides [50,68–70], as well as prebiotic lithologies such as komatiites [71] and serpentinites [52].
(2) *Metal ions/clusters in solution*: Similarly, several researchers have investigated the catalytic potential of aqueous Fe–S clusters [70,72–77]. Imai *et al.* [78] obtained hexaglycine in a thermally fluctuating solution containing Cu^2+^ ions. Cobalt aza-crown complexes have also been shown to catalyse peptide bond formation [79].
(3) *UV radiation*: Photochemistry is a recurrent theme in prebiotic synthesis (e.g. [50,80,81]). Onoe *et al.* [68] demonstrated poly-glycine formation from glycine in solution with any of several mineral-like particulates (e.g. rutile (TiO_2_), molybdenite (MoS_2_) or greenockite (CdS)) in the presence of ultraviolet radiation. UV light has also been used to generate Fe–S clusters, which in turn catalyse a variety of organic reactions ([77]; see point 2 above).
(4) *Cycles of wet/dry and thermal cycling*: Reduction of the activity of water is another theme in efforts to polymerize amino acids. The widely used salt-induced method of peptide bond formation employs wet/dry cycles of amino acids in saline solutions [82–90]. Similarly, formation of polypeptides via thermal cycling or thermal condensation has been reported by multiple groups [59,63,78,91,92].

Note that, several of these proposed mechanisms are not mutually exclusive. However, they do not explain how specific sequences with potential function ultimately emerged.

### (b) The emergence of chemical information and dynamic networks

The biological machinery of genetic inheritance preserves information in the sequences of DNA and the proteins for which it codes. The process of molecular evolution through genetic change facilitates the selection of sequences that produce functional proteins. In prebiotic environments, where peptide polymerization might occur through non-activated mechanisms described above, how might information emerge?

**Figure 2.**
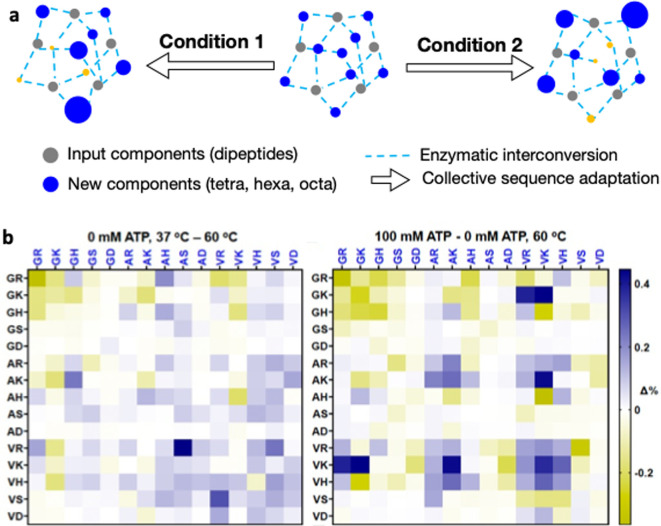
Adaptive peptide networks. (a) Populations of peptide species on a reaction network result from a balance of various supramolecular interactions. Changing the conditions shifts the ensemble over this network. (b) Networks can be represented by fingerprints, where each square is a unique tetrapeptide component formed from two input dipeptides. Absence (left) or presence (right) of ATP results in a shift in the ensemble of tetrapeptide products. Figure adapted from Kassem & Ulijn [94].

A clue to the answer comes from the field of systems chemistry. Peptidase enzymes can be used to efficiently navigate peptide self-assembly and complexation landscapes through thermodynamically driven peptide bond hydrolysis and condensation [93]. When, starting from amino acids or dipeptides, peptides of varying lengths and sequence give rise to open-ended chemical evolution. Driven by binding, self-assembly or complexation, the most kinetically and/or thermodynamically favoured sequences (or combinations of sequences) prevail as the system progresses towards equilibrium. This approach allows for the discovery of structural and functional peptides and identification of cooperative behaviours through self-selection of the most stable peptides starting from simple short building blocks (figure 2) [95]. Rich combinatorial peptide mixtures can be obtained from single and multi-component systems. The emerging peptide distribution is biased towards the peptides that have the strongest tendency to self-organize. Peptide organization and assembly is mediated by amino acid side-chain interactions, including hydrogen bonding, electrostatics and hydrophobic interactions [96]. The distribution of peptides within the mixture is correlated with the free energy stability of the emergent species under the conditions of the experiment.

These processes can efficiently self-select and amplify the synthesis of the strongest self- assembling sequences, even as long as octapeptides. For example, starting from a mixture of dipeptides containing hydrophobic and polar amino acids, out of the 100s of possible sequences that could have been formed upon exchange and oligomerization, a single oligopeptide sequence was found to be amplified over 10-fold compared to all other sequences, outcompeting all other possible combinations [97]. The system also underwent a phase change, transitioning from a homogeneous solution to a self-supporting hydrogel composed of an entangled fibre network of the self-assembled octapeptide. This experiment provides strong proof-of-concept of the role of self-organization in driving the amplification *and* selection of peptide sequences.

The next steps will be to apply a systems chemistry approach and probe how amino acid mixtures interact and collectively adapt to environmental changes relevant to the primordial Earth [97]. Also key will be to use non-enzymatic approaches and analyse outcomes at a system-level of emerging ensembles of peptides, instead of focusing on individual amplified sequences. As an example of what is possible, it was shown that one could follow the build-up and breakdown of 225 distinct interacting tetrapeptides, starting from a mixture of 15 dipeptides [97]. This experiment allowed, for the first time, the analysis of the collective behaviour of emergent dynamic networks of peptides, thus providing insights into collective peptide build-up in response to changes in external conditions—in this case, the presence or absence of ATP (figure 2). This system relies on stabilization from inter-molecular interactions with ATP to shift the equilibrium towards condensation products. In the presence of ATP, cationic tetrapeptides were amplified while neutral and anionic ones were downregulated, driven by electrostatic forces. As a result, ATP reduced the combinatorial diversity of the system [94].

Emergent peptides will likely have structures and functions that provide modes of selection such that certain peptide compositions are more likely to be more abundant in the mixture (i.e. more likely to ‘survive’) than others due to improved thermodynamic stability. Short peptide–metal chelates can vary significantly in stability [98], and metal coordination may select sequences prebiotically. Furthermore, the Lewis acidity of metals provides reactivity [9]. The presence of early-Earth-abundant metal ions such as Fe^2+^ and Co^2+^ could drive the emergence of mixtures of short peptides that perform redox chemistry, and perhaps even peptide bond ligation/hydrolysis. If this were possible, it is exciting to speculate whether amide bond formation catalysed by mixtures of metallopeptides could promote replicative autocatalytic cycles as seen in engineered laboratory systems [99–102].

### (c) Structure-guided design of pioneer peptides

Formation of specific metallopeptide sequences by spontaneous assembly and thermodynamic selection would constrain the number of possible starting catalysts from a purely random statistical mixture. The next question is which of these molecules became part of the pioneer peptides, that is which set of catalysts formed the first metabolic networks?

There are no direct molecular fossils of peptides from this time in Earth’s history. Instead we can infer their potential structure from modern enzymes using the tools of *de novo* computational protein design [103] to simplify the complex extant protein nanomachines down to the smallest peptides capable of redox catalysis. *De novo* design is a *shortcut* for studying evolution by directly optimizing the amino acid sequence for a target fold, rather than evolving it through a long series of mutations and genetic modifications. Often, during the process of design, a large number of potential sequences are generated. These can be rapidly sampled for folded, functional sequences using directed evolution, where large numbers of protein variants are generated in the laboratory and selected in a high-throughput manner [104].

**Figure 3.**
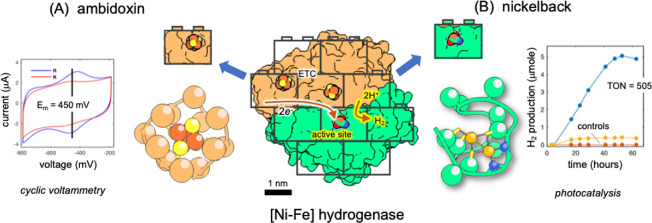
Analysis of modern nanomachines such as hydrogenase indicate a modular architecture of simpler fold building blocks. These blocks in turn can guide the structure-based design such as (A) ambidoxin—a ferredoxin mimic capable of reversible oxidation/reduction and (B) nickelback—a di-nickel binding peptide that catalyses hydrogen evolution.

One example of a successful *de novo* design approach for reducing a complex protein to a simple metallopeptide is ambidoxin (figure 3A)—an iron-sulfur binding peptide capable of reversible redox cycling. Ambidoxin is essentially the active-site loop of a broad class of electron transfer proteins: the bacterial ferredoxins. The [4Fe−4S] cubane cluster is a common inorganic cofactor mediating a wide variety of electron transfer reactions across the tree of life [105]. The ferredoxin fold binds two [4Fe−4S] cubane clusters with four cysteine residues in a highly conserved sequence motif (CxxCxxCxxxC) [106–108]. This topology is one of ten super-folds proposed to have given rise to myriad proteins [109]. The origin and evolution of the ferredoxin fold family was likely a key evolutionary milestone in origins of biological electron transfer [110]. Although the structures of ferredoxins are well studied, it initially proved challenging to design simpler peptides that stably bind iron–sulfur complexes and are capable of multiple electron transfer reactions. Several *de novo* designs based on intrinsic symmetry of iron–sulfur clusters have shown artificial folds that can bind metal, but do not exhibit reversible redox stability beyond a few dozen cycles [111–113]. Ambidoxin consists of only 12 amino acids and is highly stable.

Electron paramagnetic resonance (EPR) spectroscopy shows a characteristic rhombic spectrum indicative of a bound [4Fe−4S] cluster. Using cyclic voltammetry (an electrochemical method for measuring current with varying voltage potential), it was found that ambidoxin would survive thousands of successive oxidation/reduction cycles. This incredible redox stability matches and in some cases exceeds the performance of its modern, evolved counterparts [114].

Another pioneer peptide recapitulates the active site of [Ni–Fe] hydrogenase. This nickelback peptide is coordinated to two nickel ions within a 13-amino acid sequence and catalyses hydrogen evolution (2H^+^ + 2e^−^ ⇌ H_2_) [115] (figure 3B). Nickelback binds two Ni(II) ions coordinated by four evenly spaced cysteines and two backbone amides [115]. Soluble nickel was far more abundant in the Archean ocean than it is today [9,116], making it potentially available for emerging peptide catalysts. The nickelback sequence was derived from the analysis of structures of hydrogenases which catalyse oxidation/reduction of hydrogen [117,118] and acetyl-CoA synthetases [119] from the ancient carbon-fixing Wood–Ljungdahl pathway [120]. Nickelback undergoes robust, reversible oxidation and reduction under a wide range of solution conditions. A clear catalytic current peak indicates nickelback can catalyse a reduction reaction, producing H_2_ with 500 to 1000 turnovers (repeated catalytic cycles) per molecule of enzyme. As with ambidoxin, nickelback showed high stability and robust activity despite, or perhaps because of its simple architecture.

Our experimental results strongly suggest that the first epoch in the emergence of metabolism may have been the thermodynamic selection of short metallopeptides from free amino acids and transition metals in primordial environments. These, or similar, short peptides would have been the pioneers of redox chemistry at the origin of life.

The performance of laboratory models of putative pioneer peptides challenges the widely held notion that early proteins were inefficient, unstable catalysts that required extensive optimization through genetic evolution. *What if the first peptides were highly stable and highly active, providing a key energetic advantage to early organisms?* That would make them compelling pioneer enzymes that could subsequently evolve into modern nanomachines that power life. Still, we do not know how such peptides were incorporated in the first translation machinery—that is how did replicative and energetic systems find each other? There is much still to be learned about this first epoch in the emergence of metabolism.

## 3. Epoch 2—early protein folds

If short metallopeptides constituted the foundation of metabolism, what drove their subsequent complexification into protein folds? Proteins adopt a multitude of three-dimensional folds, composed largely of periodic chain structures—α-helices and β-sheets—connected by loops. While over half of the protein participates in interactions which served to stabilize the fold, it is generally the loops where the most interesting, functional interactions occur. Sites involved in molecular recognition or catalysis are often mediated by one or more loops. These sites of molecular interaction often contain a small number of residues that have evolved to cooperate to stability interactions [121]. Short peptides such as nickelback and ambidoxin are essentially all-loop, with thermodynamic stability coming from the metal-peptide coordination bonds. In the case of larger proteins, the burden of stability can be transferred to the fold, allowing the active sites to evolve and diversify function. Larger proteins support long-range dynamics that can be essential in modulating function through allosteric interactions. The path of bacterio-ferredoxin evolution from peptide to protein is providing insight into the driving factors behind complexification.

### (a) Ferredoxins are ancient proteins

The bacterio-ferredoxins (herein referred to simply as ferredoxins) are thought to be one of the earliest proteins. The ambidoxin peptide was designed based on the active site loops of ferredoxins. Several features point to their ancient origins. Ferredoxins are iron–sulfur (Fe–S) binding oxidoreductases that are present across all kingdoms of life [122–124]. They are relatively small proteins (60 amino acids long) and fold as a tandem pair of β–α–β secondary structure elements. They coordinate two cubane [4Fe−4S] clusters [75,125,126]. Each cluster is coordinated through four highly conserved cysteines as first shell ligands [110,127]. Ferredoxins typically function as low-potential (−260 to −680 mV versus standard hydrogen electrode) single-electron carriers [128,129], matching geochemical redox couples abundant in the early Archean Earth and in modern reducing environments [10,106,130,131]. Iron–sulfur complex chemistry in the absence of proteins has been implicated in prebiotic metabolic reactions [77,132]. Today, ferredoxins are found in diverse bioenergetic pathways from methanogenesis and photosynthesis to nitrogen fixation and aerobic respiration [106,133–136]. They shuttle electrons within the cell between donor and acceptor redox proteins, or are embedded as domains within larger oxidoreductases, acting as wires sometimes spanning several nanometres within a protein [133,137,138]. The simple fold, redox energetics and ubiquity in metabolism are consistent with the ancient origin of the ferredoxins. Ferredoxins serve as a model for investigating the transition from small peptides to protein folds [122].

### (b) The Dayhoff–Eck hierarchy

**Figure 4.**
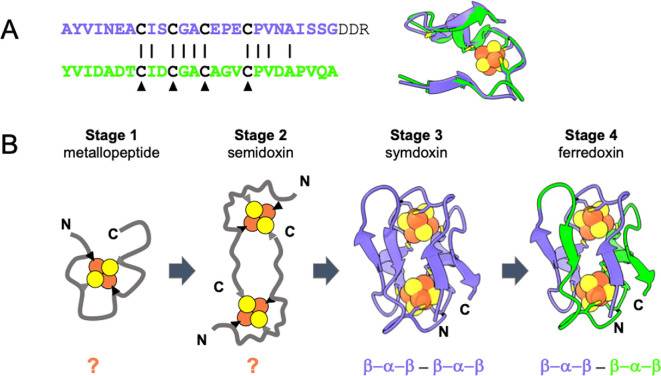
Dayhoff’s hierarchy provides a framework for studying ferredoxin evolution. (A) Homology evidence of repeated sequence and two-fold structural symmetry of a natural ferredoxin (sequence and aligned domains of *C. acidurici* ferredoxin [139]). (B) Ferredoxin evolution based on the original Dayhoff proposal [122]. Little is known about the structure and function of the first two stages. Duplication and fusion of two semidoxins likely generated full-length symdoxins that diversified into extant ferredoxins.

Sequence and structural symmetries in ferredoxin suggest an evolutionary trajectory that extends deep into life’s history, perhaps back to a prebiotic–biotic emergence of metabolism. The two β–α–β domains of ferredoxin have sequence homology and are structurally equivalent, related by two-fold cyclic symmetry (*C_2_*), each domain binding one [4Fe−4S] cluster (figure 4) [140]. This architectural symmetry has evolutionary implications, first noted by Eck and Dayhoff nearly 60 years ago [141]. They proposed a hierarch trajectory of complexity from: *stage 1*: short metal-binding peptides; *stage 2*: around 30 amino acids long β–α–β ferredoxin halves; *stage 3*: around 60 amino acids long (β–α–β)–(β–α–β) ferredoxins formed through genetic duplication and fusion; to *stage 4*: evolution of extant ferredoxins diversification of the N- and C-terminal halves. Dayhoff’s hierarchy provides a concrete, testable framework for modelling the deep-time evolution of the ferredoxin fold.

Stage 1 of Dayhoff’s hierarchy has been explored by several laboratory models. Short peptides, including ambidoxin, can bind [4Fe−4S] clusters and in some cases reversibly cycle between oxidized and reduced states [113,142–145]. They can function as electron carriers, driving formation of a pH gradient in protocell models [146]. These simple systems suggest a plausible connection between genetically encoded iron–sulfur proteins and spontaneously assembling metallopeptides during the emergence of metabolism [13,98,147,148].

The transition to stage 2 reflects a critical stage in the evolution of proteins and must involve the machinery of genetic inheritance—DNA, RNA with their associated polymerases and a ribosome that can translate nucleic acid sequences into amino acids. It is really the transition from prebiotic chemistry to biochemistry. The syndiotactic, heterochiral ambidoxin is unlikely a direct ancestor to the ‘semidoxin’—a short, 30 residue half-ferredoxin. Intermediates in this prebiotic-life transition may be too long for systems chemistry to elucidate and will likely need to be addressed by *de novo* protein design approaches to model this stage. As previously indicated, there is a critical gap here—the integration of metabolic peptides and replicative nucleic-acid-based systems—where concrete, experimental models of such a transition are lacking, with a few notable exceptions [149].

Stage 3 of Dayhoff’s hierarchy posits a fully symmetric, single-chain ancestor of extant ferredoxins. This stage has been modelled using ancestral sequence reconstruction, indicating increasing sequence identity between N- and C-terminal halves at branches approaching the root of the ferredoxin tree [141]. Computational structure-guided designs of symmetric ferredoxins could express and assemble with [4Fe−4S] clusters *in vivo* and could function as electron carriers in an engineered pathway [150,151]. Such symmetric ferredoxins could represent a potential evolutionary bridge between metallopeptides and modern ferredoxins.

The growth of peptides into proteins may be at the root of a large number of protein folds [152]. Soding and Lupas propose a ‘vocabulary’ of ancient peptides, that recur in modern proteins and are repeated or combined as modular units to build the ‘galaxy’ of fold-types in extant proteins [153]. The Dayhoff–Eck hierarchy is an example of how such ancient peptides may have evolved into folds. Experimental demonstrations of these intermediates in evolution are being undertaken for not only ferredoxins, but also the nucleotide binding folds [154,155], showing the stability and functionality of lost transition fossils as proteins traversed the gap from prebiotic chemistry to life.

## 4. Epoch 3—protein nanomachines and complex life

Protein folds, or domains, are a fundamental unit of molecular evolution in all extant life on Earth [14]. Complex proteins seldom emerge *de novo* but are instead formed by the duplication and recombination of existing domains. Just as amino acids are the building blocks of peptides and secondary structural units such as helices and sheets are building blocks for folds, the folded domains are the building blocks of many of the massive protein nanomachines involved in metabolism [106,156]. Photosystems I and II [157,158], nitrogenase [159], the hydrogenases [118], RuBisCO [160] are all giant proteins comprising multiple smaller domains. Multiple bacterial ferredoxins integrated into a single protein can form a chain of Fe–S clusters capable of transferring electrons along a protein wire over tens of nanometres [133]. Similar concatenations of heme-binding cytochrome domains form cell-scale wires that contact metal surfaces and allow microbes, such as those in the genera *Geobacter* or *Shewanella*, to breathe electrons [161,162]. The complexity of an organism is matched by the complexity of its proteins [163], with terrestrial plants and metazoans having the largest, most complex multi-domain proteins of any kingdom in the tree of life. The third and most recent epoch in protein evolution is the assembly of domains into complex metabolic protein nanomachines.

### (a) Identifying the building blocks

Even though there are countless proteins across life, there are likely only *ca* 400 core gene groups responsible for redox metabolism [1,3], which can be clustered based on homology of their amino acid sequences. However, these 400 groups are largely complex, multi-domain protein nanomachines. One powerful approach to study the history of proteins is phylogenetics, a statistical method for reconstructing evolutionary relationships using the DNA or protein sequence. However, modern phylogenetic tools cannot be used readily to infer evolutionary processes spanning billions of years. Instead, given that the three-dimensional folds of proteins change more slowly than their sequences [164,165], structure-based approaches allow us to look deeper in time and infer protein ancestry [106,130,166–169]. Machine learning approaches applied to protein structure prediction [170,171] have expanded databases from hundreds of thousands [172] to hundreds of millions of structures [173] in just a few years, vastly increasing the statistical power to make evolutionary inferences [171].

**Figure 5.**
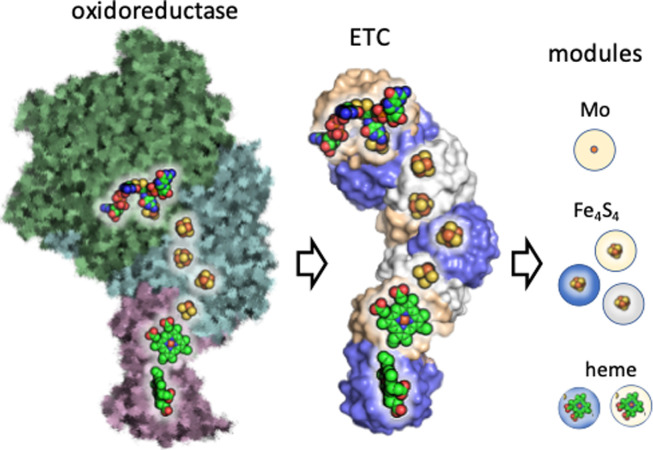
Modular architecture of complex enzymes. Many oxidoreductases are large nanomachines. Functioning at the core of these proteins are electron transport chains (ETCs) where electrons move through transition metals held by the protein matrix. The protein folds around the metals were clustered into a few recurring groups of folds, which represent ancient modules of oxidoreductases.

Given the potential of using protein structure to probe deep-time evolution, how could one narrow down which folds likely had their origins in the earliest enzymes? Based on the observation that protein structures are more conserved within a nanometre of metal binding sites [169], one approach compared protein structures using only the neighbourhood of amino acids surrounding the active site (figure 5). Despite the thousands of potential folds available, just a handful were found at the centre of redox enzymes [106]. Many of these core folds were already proposed as ancient in origin by similar approaches [166–168,174]. These building blocks likely had their origins in ancient folds that alone were catalytically active but could be modularly assembled to produce complex enzymes and metabolic reaction networks.

### (b) Deep-time domain evolution

Domain classification schemes identify anywhere from hundreds to thousands of unique folds, depending on the selection criteria. Would we imagine that each of these folds has an independent origin from a pioneer peptide? Alternatively, a handful of protein folds could have first emerged, and then evolved and diversified to produce the modern vocabulary of domains. This is a challenging question to answer, as we currently lack fold-similarity metrics that have a foundation in evolutionary theory similar to the molecular clock hypothesis for sequence similarity that is the foundation for phylogenetics. Therefore, it is challenging to specify whether related folds are connected by ancestry or convergence.

**Figure 6.**
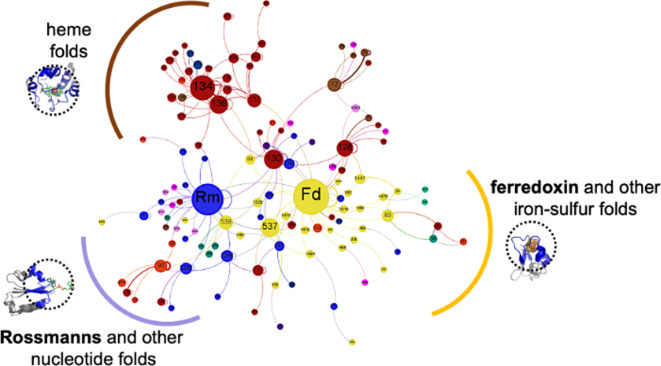
Wiring diagram of protein domains suggests deep-time evolutionary connections. Oxidoreductases were divided into modular building blocks (*nodes*). Spatial connections between blocks (*edges*) show possible paths for electrons to move through proteins and drive biochemical reactions. A network representation of all block–block connections shows neighbourhoods of iron–sulfur (yellow), heme (brown) and nucleotide (blue) modules and connecting edges which are paths for electrons. Curved self-connecting edges represent the evolutionary process of domain duplication. We hypothesize that edges between different blocks might indicate deep-time duplication and extreme diversification.

In the case of metabolic proteins, particularly the large nanomachines, there is additional information in the structures that has provided some insight into whether the folds of metabolism originate from a few ancient building blocks, or not. Within oxidoreductases, metal centres form molecular highways that move electrons through the protein matrix. This topology allows the generation of a network of putative electron transfer paths between folds, that is a literal biological ‘wiring diagram’ of the electrical connections between building blocks [106,130]. Surprisingly, folds that were connected to each other in this network often bound similar redox cofactors, leading to communities within the network of folds that bind iron-sulfur, heme, copper, nucleotides, and so on (figure 6). These similarities suggest a framework for exploring protein evolution where folds connected in protein wires may be evolutionarily related through a process of gene duplication of a cofactor-binding domain and extensive diversification to the point where only cofactor coordination is preserved. Wires could reflect deep-time evolutionary connections that would not be evident by phylogenetics nor structure-based evolutionary approaches. Each edge in this network is a potential evolutionary transition that happened billions of years ago.

### (c) A shared origin of two folds

At the centre of this wiring diagram is not one, but two folds—the bacterial ferredoxin and the Rossmann fold. Like ferredoxins, Rossmann folds are also likely ancient [175–178]. The Rossmann fold typically coordinates nucleotide phosphate groups, but is also found in nitrogenase and hydrogenase coordinating the large iron–sulfur metallocofactors [176]. Rossmann folds consist of a four-stranded parallel β-sheet, flanked by α-helices, with phosphates or metalloclusters interacting with the carboxy-terminal end of the sheet near a ‘crossover’ position at the crown of the domain. The cluster ligands are, in turn, positioned in the loops between the different strands.

Phosphate binding is essential in regulating life processes [179], and it has been suggested that the first phosphatase likely emerged at the transition from the RNA to the primordial RNA–protein world [180]. Further, phosphate binding is ubiquitous in biology; nearly half of all known protein structures (as of 2011) were demonstrated to interact with phosphate-containing ligands [181,182]. Two particular phosphate binding motifs stand out: the Walker A (GxxxxGK(S/T) and Rossmann (GxxGxGR(S/T)) P-loops. Notably, both Walker A and P-loop motifs occur between the first β-strand and the α-helix in the proposed last universal fold ancestor (LUFA). These loops are both glycine-rich motifs, with similar sequence patterns [154]. However, despite these similarities, there are also some significant differences between the Rossman and Walker-A loops. Specifically, the Rossmann loops typically use the bound phosphate group as a binding handle, whereas the Walker-A P-loops (which are typically present in ATP or GTP binding proteins [183–186]) facilitate chemistry on the bound phosphate group [154], recruiting a metal ion (usually Mg^2+^) for this purpose. The evolution of the cation binding site in P-loop nucleoside triphosphatases has, in turn, been suggested to be important for the evolution of the basic catalytic mechanism of these enzymes [187].

**Figure 7.**
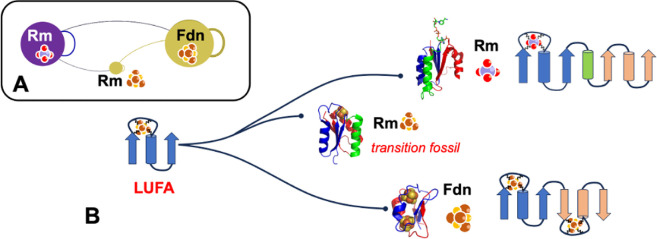
Proposed evolution of LUFA into ferredoxin and Rossmann folds. (A) Electron transfer pathway connections between Rossmann (Rm) and ferredoxin (Fdn) folds observed in the oxidoreductase wiring network (see figure 6). This pathway included a secondary Rossmann-like fold bound to iron–sulfur ligands. (B) Hypothesized LUFA is duplicated to form ferredoxin or duplicated with an additional helix (green) to form the Rossmann folds. The iron–sulfur binding Rossmann may be the transition fossil from LUFA to the nucleotide-binding Rossmann.

Could the two folds share a common ancestor? Proteins in the ferredoxin and Rossmann fold groups perform ancient chemistries essential for life. There are numerous examples in oxidoreductases where ferredoxins and Rossmann domains are wired together. Sometimes, the Rossmanns bind an [4Fe−4S] cluster [176], rather than a nucleotide, forming an electron transfer pathway with adjacent ferredoxins [130] (figure 7A). One interpretation is that this reflects fossil evidence of duplication and extensive structural diversification. The metal-binding Rossmanns may be a transition fossil between ferredoxins and nucleotide binding Rossmanns. Analysis of the topology of α-helices and β-sheets in the two folds indicates ferredoxin and Rossmann folds could interconvert through the insertion/deletion of a short α-helix between two β−α−β domains [130] (figure 7B). The repetition of this core topology in both folds drives the core question of whether an ancestral fold unifies these two major groups.

Whether the domains that have facilitated the third epoch of metabolic protein evolution share a common origin is very challenging to test. One approach may be to identify modern examples of fold-switching [188], cases where proteins can switch between two conformations through small genetic perturbations. An engineered example of a fold-switching protein shows that one can switch from a mixed α/β fold to an all α-helical protein by a single mutation [189]. Whether such events are common or rare in natural protein evolution will help us better estimate the plausibility of a common-origin model. Similarly, using protein design, it may be possible to recapitulate these early transitions, for example, by designing sequences that can switch between a ferredoxin and Rossmann fold with minimal changes.

## 5. Future questions

Finally, machines move. Although we have atomic level structures for many core metabolic nanomachines, we do not yet understand how they originated, let alone how they work. This lack of understanding is especially obvious in at least two critical oxidoreductases: Photosystem II, which is the machine that splits water from light, and nitrogenase, the machine responsible for generating NH_4_^+^ from N_2_ gas. Many additional nanomachines are responsible for energy transduction and metabolism, however two stand out: the origin and evolution of the ribosome, the machine responsible for making proteins, and the rotary ATPases, the machines responsible for generating cellular energy. All these core nanomachines are highly dynamic, that is, they functionally move at the molecular level to catalyse their respective reactions. While there is much to be learned about the origin and evolution of life, fundamentally, we do not understand the rules for the emergence of functional dynamics in core proteins responsible for metabolism.

In this perspective, we describe how systems chemistry and comparative analysis of three-dimensional protein structure has extended our understanding of deep-time evolutionary events. Large-scale global motions in proteins are often essential for function [190–192], and are robust against random mutations [193]. Perhaps such motions are preserved over longer timescales than structure or sequence and the application of comparative dynamics approaches could take us further back in time.

## Data Availability

This article has no additional data.
